# Laser microdissection coupled with RNA-seq analysis of porcine enterocytes infected with an obligate intracellular pathogen (*Lawsonia intracellularis*)

**DOI:** 10.1186/1471-2164-14-421

**Published:** 2013-06-24

**Authors:** Fabio A Vannucci, Douglas N Foster, Connie J Gebhart

**Affiliations:** 1 Department of Veterinary and Biomedical Science , College of Veterinary Medicine, University of Minnesota, St. Paul, MN 55108, USA; 2 Department of Animal Science, College of Food, Agricultural and Natural Resource Science, University of Minnesota, St. Paul, MN, USA

**Keywords:** Microdissection, RNA-seq, Obligate intracellular, *Lawsonia intracellularis*, Proliferative enteropathy

## Abstract

**Background:**

*Lawsonia intracellularis* is an obligate intracellular bacterium and the etiologic agent of proliferative enteropathy. The disease is endemic in pigs, emerging in horses and has been described in various other species including nonhuman primates. Cell proliferation is associated with bacterial replication in enterocyte cytoplasm, but the molecular basis of the host-pathogen interaction is unknown. We used laser capture microdissection coupled with RNA-seq technology to characterize the transcriptional responses of infected enterocytes and the host-pathogen interaction.

**Results:**

Proliferative enterocytes was associated with activation of transcription, protein biosynthesis and genes acting on the G_1_ phase of the host cell cycle (Rho family). The lack of differentiation in infected enterocytes was demonstrated by the repression of membrane transporters related to nutrient acquisition. The activation of the copper uptake transporter by infected enterocytes was associated with high expression of the Zn/Cu superoxide dismutase by *L. intracellularis*. This suggests that the intracellular bacteria incorporate intracytoplasmic copper and express a sophisticated mechanism to cope with oxidative stress.

**Conclusions:**

The feasibility of coupling microdissection and RNA-seq was demonstrated by characterizing the host-bacterial interactions from a specific cell type in a heterogeneous tissue. High expression of *L. intracellularis* genes encoding hypothetical proteins and activation of host Rho genes infers the role of unrecognized bacterial cyclomodulins in the pathogenesis of proliferative enteropathy.

## Background

Cell proliferation concomitant with bacterial infections has been associated with carcinogenesis in chronic diseases caused by *Helicobacter pylori, Salmonella typhi* and *Citrobacter rodentium*[1]. Proliferative changes resulting in a hyperplastic but non-carcinogenic process is induced in *Lawsonia intracellularis* and *Bartonella* spp. infections [1]. The inflammatory mediators generated during the chronic gastritis in *H. pylori* infections have been related with oxidative DNA damage and cell transformation [2,3]. However, other gram negative pathogenic bacteria (e.g. *L. intracellularis* and *C. rodentium*) are able to promote enterocyte proliferation with minimal inflammatory responses [4,5]. *C. rodentium* induces proliferation of mouse colonic enterocytes when it attaches to the apical membrane [6]. It is believed that *L. intracellularis* escapes from the endosome after internalization and multiplies freely in the cytoplasm of undifferentiated intestinal crypts promoting their proliferation and progressive replacement of the differentiated intestinal epithelium by immature infected enterocytes. Although early pathogenesis studies also reported the ability of *L. intracellularis* to infect mature enterocytes, the bacterium seems to establish the infection in crypt cells [7]. These unusual pathological changes characterized by the presence of a large number of intracellular bacteria and proliferation of enterocytes suggests that *L. intracellularis* has adopted mechanisms of survival and pathogenesis that are unique among bacterial pathogens [5]. To date, hypotheses and speculations have been discussed regarding the pathogenesis of this infection but, the underlying mechanisms by which *L. intracellularis* induces proliferative changes have not been addressed.

*Lawsonia intracellularis* is an obligate intracellular bacterium and the etiologic agent of proliferative enteropathy (PE) [8]. Mild to severe diarrhea is the major clinical sign described in infected animals and is directly associated with cell proliferation and replication of the bacteria in the intestinal epithelium [9]. PE is endemic in swine herds, an emerging disease in horses and has been reported in various other species, including non-human primates, wild mammals and ratite birds [10-13]. Microscopically, different levels of hyperplasia in the intestinal crypts associated with significant reduction in the number of goblet cells are observed in animals affected with PE. Although inflammation is often observed at late stages of the disease, it does not represent a primary characteristic associated with *L. intracellularis* infection.

The disease was first reported in 1931 but, due to its fastidious properties, porcine *L. intracellularis* was first isolated in 1993 using rat small intestinal cells cultured in a strictly defined microaerophilic environment [14,15]. Since then, the chronological dynamics of infection *in vitro* and *in vivo* have been well-characterized [16,17]. While proliferative changes *in vivo* follow an increase in the number of intracellular bacteria, the bacterium is not able to induce proliferation in infected cells *in vitro*[18]. This intriguing observation associated with the fastidious properties of this bacterium has limited the studies on the pathogenesis of *L. intracellularis*. Additionally, the adaptation of this microaerophilic, but obligate intracellular organism to grow freely in the cytoplasm of metabolically active enterocytes suggests that this organism has properties that are novel and unique among bacterial pathogens.

In the present study, we hypothesized that genes expressed by *L. intracellularis* in the host cytoplasm are capable of inducing proliferation and preventing differentiation of immature enterocytes by altering cell cycle-associated pathways. We established a method integrating laser capture microdissection (LCM) and RNA-seq technology to characterize the host gene expression and the host-bacterial interactions *in vivo*. Activation of transcription, protein synthesis and Rho family genes in infected enterocytes characterized the transcriptional mechanisms involved in the cell proliferation. The ability of *L. intracellularis* in preventing enterocyte differentiation and maturation was proved by the consistent down-regulation of apical membrane transporters related to nutrient acquisition by infected enterocytes. The intracellular bacteria showed high level of expression of a sophisticated oxidative protection mechanism which involves redox enzymes and a rubrerythrin-rubredoxin operon (*rubY*-*rubA*). Rho genes expressed by the host and associated with the high expression of bacterial genes encoding hypothetical proteins implies a potential role of unrecognized bacterial effector proteins that modulate eukaryotic cell cycle (cyclomodulins) in the pathogenesis of PE.

## Results

### Technical characterization of the LCM coupled with RNA-seq analysis

Based on previous studies reporting the chronological course of the *L. intracellularis* infection *in vivo*[19,20], experimentally-infected pigs in our study were monitored every other day regarding clinical signs (diarrhea and general attitude) and quantitative fecal shedding of *L. intracellularis* DNA until the end point of the study (21 PI). The negative control group was also monitored and its negative status was consistent throughout the study period. One animal from the infected group exhibiting only mild to moderate lesions typical of PE associated with grade 1 and 2 in the IHC grading scheme was discarded from the experiment. In order to have the same number of replicates in both infected and control groups, one animal was randomly discarded from the negative control group. As a result, five biological replicates (animals) from each group were included in the LCM procedures and RNA-seq analysis.

**Figure 1:**
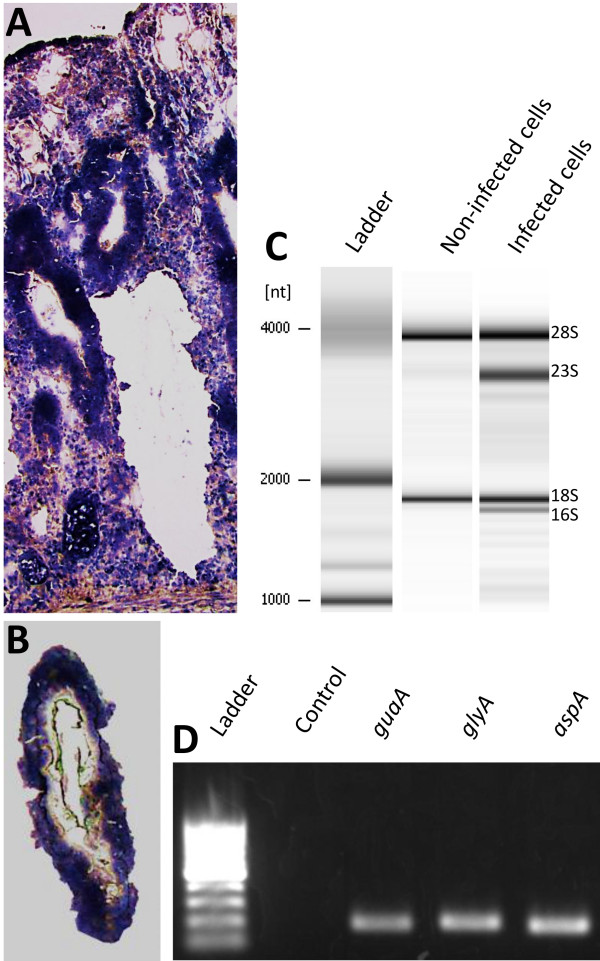
**Laser capture microdissection of an intestinal crypt infected with *L. intracellularis *(A-B) and evaluation of RNA quality from microdissected cells (C-D). (A)** Hematoxylin stained cryosection of infected ileal mucosa. **(B)** Microdissected intestinal crypt captured in the thermoplastic film of the LCM cap prior to RNA isolation. **(C)** Agilent Bioanalyzer data showing bacterial and eukaryotic ribosomal RNA in infected and non-infected cells. **(D)** One-step RT-PCR products of three protein-encoding genes of *L. intracellularis*.

The quality of RNA samples extracted from microdissected tissues was evaluated by assessing bacterial and eukaryotic ribosomal RNAs using Agilent Bioanalyzer 2100 (Figure 1C). The successful recovery of high quality eukaryotic RNA from microdissected cells using the LCM procedures described in our study has been well-established [21]. Since studies using prokaryotic RNA from microdissected cells have not been well-explored, the feasibility of using *L. intracellularis* mRNA for sequencing was confirmed by one-step RT-PCR using specific primers targeting three housekeeping genes (Figure 1D).

The sequence reads representing the RNA transcripts derived from the host cells and the intracellular bacteria were mapped onto both pig (*S. scrofa* 10.2) and the *L. intracellularis* (PHE/MN1-00) reference genomes available at NCBI. From a total average of 22,136,064 reads generated, 82% (18,280,138) and 4% (1,000,440) were mapped against the porcine and the bacterium genome, respectively (Additional file 1: Table S2). A total of 11,778 genes were expressed in the porcine genome and met the criteria for differential expression analysis (see Materials and Methods). Up-regulation of 119 and down-regulation of 46 protein coding genes were identified in infected enterocytes (fold-change ≥2.0; *p*<0.05) (Additional file 2: Table S3).

In the bacterial gene expression analysis, 754 protein coding genes had at least one mapped read against the reference DNA sequence of the *L. intracellularis* PHE/MN1-00 isolate, which was the same isolate used in the experimental infection. The top 20 genes highest expressed by *L. intracellularis* and their respective normalized transcription levels based on the RPKM values are described in Table 1.

**Table 1:** **The top 20 highly expressed genes by *L. intracellularis *in the enterocyte cytoplasm**

**Locus**	**Gene product**	**Predicted function**	**Log**_**2 **_**(RPKM)**
LI0461	Hypothetical protein	Unknown	15.0
LI0625	Chaperonin GroEL	Protein folding	14.9
LI1159	Hypothetical protein	Unknown	13.7
LI0447	Hypothetical protein	Unknown	13.6
LI0935	Elongation factor Tu (*tufA*)	Protein biosynthesis	13.5
LI0624	Co-chaperonin GroES	Protein folding	13.5
LI0005	Cu-Zn superoxide dismutase precursor (*sodC*)	Oxidative stress	13.3
LIC060	Hypothetical protein	Unknown	12.9
LI0267	Hypothetical protein	Unknown	12.8
LI1075	Dioxygenases related to 2-nitropropane dioxygenase	Oxidative stress	12.7
LI0809	Hypothetical protein	Unknown	12.5
LI0912	Molecular chaperone DnaK	Protein folding	12.4
LI0439	Hydrogenase-1 small subunit	Cell metabolism	12.4
LI0902	Outer membrane protein related to OmpA-OmpF porin	Membrane transport	12.4
LI1158	Hypothetical protein related to secretion system effector C	Intracellular survival	12.3
LI1005	Pseudouridine synthase (*truB*)	Protein biosynthesis	12.2
LI0559	50S ribosomal protein L13	Protein biosynthesis	12.2
LI0697	Rubrerythrin (*rubY*)	Oxidative stress	12.2
LI0043	Hypothetical protein	Unknown	12.0
LIC103	Methyl-accepting chemotaxis protein (*pilJ*)	Chemotaxis	11.9

In order to screen for the presence of any other microbial organisms in the experimental samples, the sequence reads were also mapped against viral, bacterial and archaeal genome databases. While no detectable hits were found against the viral and archaeal genomes, 0.052% and 0.084% of the sequence reads from the infected and control group, respectively, were mapped against the bacterial database. Three unculturable bacterial species (*Candidatus Zinderia*, *Candidatus Carsonella ruddii*, *Candidatus* Sulcia muelleri) and *Propionibacterium acnes* were commonly found in both groups. An uncharacterized species of the genus *Peptoniphilus* previously reported in the oral cavity of a human was exclusively found in the control group.

**Figure 2:**
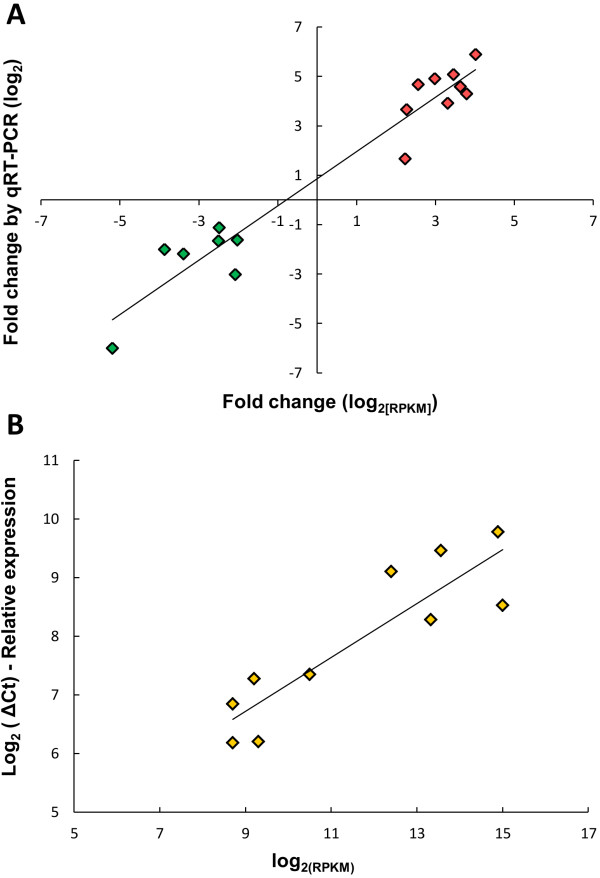
**Correlation between RNA-seq and qRT-PCR expression data. (A)** Plot of the relative fold-change in gene expression of 16 porcine genes from infected enterocytes by RNA-seq (x-axis) and qRT-PCR (y-axis). R^2^ = 0.92 (*p*<0.05). **(B)** Plot of the relative quantification of ten *L. intracellularis* genes by RNA-seq (x-axis) and the transcript levels generated by qRT-PCR (y-axis). R^2^ = 0.81 (*p*<0.05).

The RNA-seq expression data were validated by qRT-PCR based on the relative quantification of 16 differentially expressed genes from the host and 10 bacterial genes (Additional file 3: Table S1). The averages of fold-change in gene expression from infected enterocytes and the relative transcriptional levels of *L. intracellularis* genes were plotted against the log_2_ (RPKM) (Figure 2). The log_2_ transformed fold-change from the host gene and transcript levels from the bacterial genes were positively correlated on a linear regression model (*p*-value < 0.05). Based on the qRT-PCR validation, the RNA-seq data properly estimated the fold-change expression in infected enterocytes and the transcription levels of *L. intracellularis* genes.

### Protein biosynthesis and transcription

**Figure 3:**
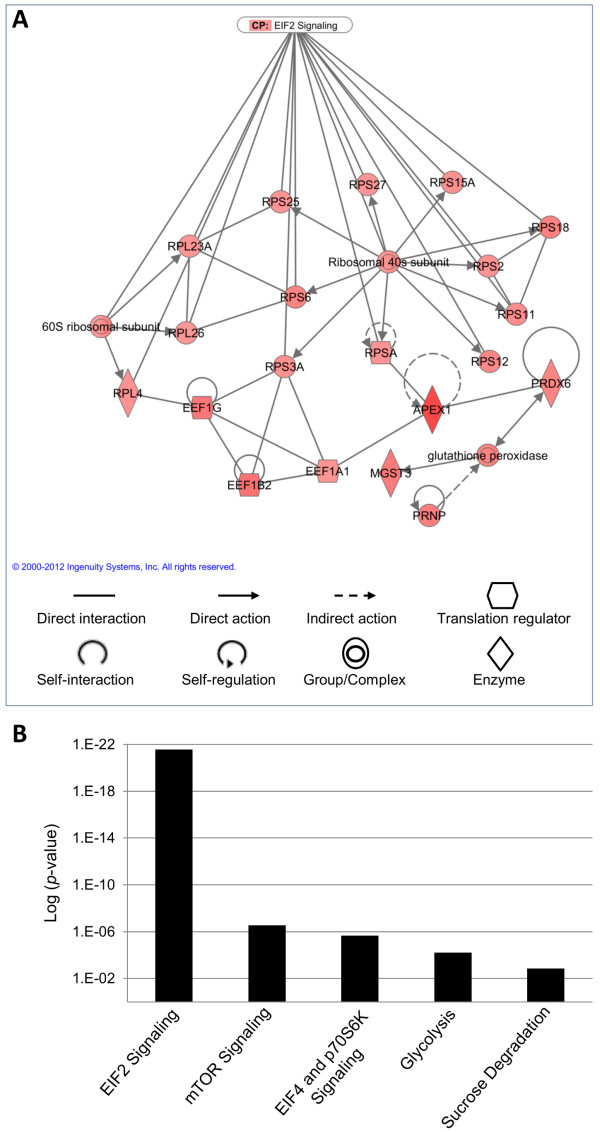
**Cellular network and canonical pathway analysis of genes differentially expressed by enterocytes infected with *L. intracellularis*. (A)** Molecular interaction representing part of the protein biosynthesis network which was most correlated with the set of genes significantly up-regulated in infected cells. Strong interaction between the main canonical pathway (CP: EIF2 signaling) identified in the differentially expressed genes and the protein biosynthesis network. **(B)** Canonical pathways (x-axis) most associated with genes differentially expressed based on the -log of *p*-value calculated by Fisher's exact test (y-axis).

The 165 porcine genes differentially expressed in *Lawsonia*-infected enterocytes were analyzed regarding their biological functions and molecular networks based on the mammalian gene expression information available in the Ingenuity® System. The system knowledge database recognized and analyzed 144 differentially expressed genes. The IPA system associates the set of differentially expressed genes with cellular networks (focus genes) and creates a score based on the number of network eligible genes they contain (Table 2). The protein biosynthesis network was most correlated with the set of genes differentially expressed and up-regulated in *Lawsonia*-infected enterocytes. Figure 3A shows the molecular interaction of this network which mainly includes ribosomal proteins and mRNA translation factors. The eukaryotic initiation factor (IEF2) signaling was the canonical cell pathway (CP) more significantly associated (*p*-value 2.76E-22) with the genes differentially expressed (Figure 3B). A remarkable influence of IEF2 signaling on the protein biosynthesis was identified by merging both network and pathway analysis (Figure 3A).

**Table 2:** **The four cell networks most associated with the genes differentially expressed in *Lawsonia*-infected enterocytes**

**Associated network functions**	Score*	**Focus genes**
Protein Synthesis, Cellular Assembly and Organization, Molecular Transport	59	27
RNA Post-Transcriptional Modification, Cancer, Hematological Disease	42	21
Cellular Movement, Cancer, Reproductive System Disease	31	17
Cell Death and Survival, Carbohydrate Metabolism, Molecular Transport	31	18
* Based on the number of network eligible genes within the referred network.		

Functional clustering analysis using DAVID knowledge database confirmed the involvement of the 27 differentially expressed genes in the protein biosynthesis (data not shown) and also showed the association of four genes acting in the positive regulation of transcription (Table 3). The activation of these two cellular processes (transcription and protein biosynthesis) revealed a global increasing in the cell metabolism in response to the *L. intracellularis* infection and it has also been described to occur during the gap phase 1 (G_1_) of the host cell cycle [22].

**Table 3:** **Functional clustering associated with genes differentially expressed in *Lawsonia*-infected enterocytes**

**Gene Symbol**	**Gene product**	**ln(fold change)**
***Clustering***		
*Positive regulation of transcription*		
TEF1	Transcriptional enhancer factor 1	2.2
SMARCC2	SWI/SNF complex subunit SMARCC2-like	2.3
SOX-9	Transcription factor SOX-9	2.6
FHL2	Four and a half LIM domains 2	2.1
*Mitotic cell cycle-associated genes*		
CDK2	Cyclin-dependent kinase 2	2.6
RHOA	Ras homolog family member A	2.2
RHOB	Ras homolog family member B	2.2
RRAGA	Ras-related GTP-binding protein A	2.3
RPL24	60S ribosomal protein L24-like	2.7
SKA2	Spindle and kinetochore-associated protein 2-like	2.1
PSMA1	Proteasome subunit alpha type-1-like	2.4
*Pro-apoptosis-related genes*		
SLAM7	Signaling lymphocytic activation molecule 7	−4.6
C6	Complement component C6	2.3
C9	Complement component C9	3.5
PRDX1	Peroxiredoxin 1	2.2
SST	Somatostatin	−3.4
TNFSF10	Tumor necrosis factor ligand 10	−2.5
VAV2	Vav 2 guanine nucleotide exchange factor	−2.1
*Anti-apoptosis-related genes*		
DAD1	Defender against cell death 1	2.3
NME2	Non-metastatic cells protein	2.9
PRNP	Prion protein	2.6
PPP2CB	Protein phosphatase 2, catalytic subunit, beta isozyme	2.1
RHOA	Ras homolog family member A	2.2
TPT1	Translationally-controlled tumor protein-like	2.7
ERBB3	Receptor erythroblastic leukemia viral oncogene	2.2

### Cell cycle and apoptosis

In addition to the positive regulation of transcription, DAVID clustering analysis identified genes associated with cell cycle and apoptotic events differentially expressed in infected enterocytes (Table 3). Cell cycle-associated genes mainly represented by Ras homolog proteins were significantly induced in association with Cyclin-dependent kinase 2 (CDK2). The aberrant activation of Rho-genes including those described in our study has been well-described in oncogenesis by causing deregulation of cell cycle progression and promoting cell proliferation [23]. Additionally, the co-expression of Rho proteins and Cyclin-dependent kinases (CDKs) specifically act by stimulating the entry and progression of the G_1_ phase of the host cell cycle [24].

**Figure 4:**
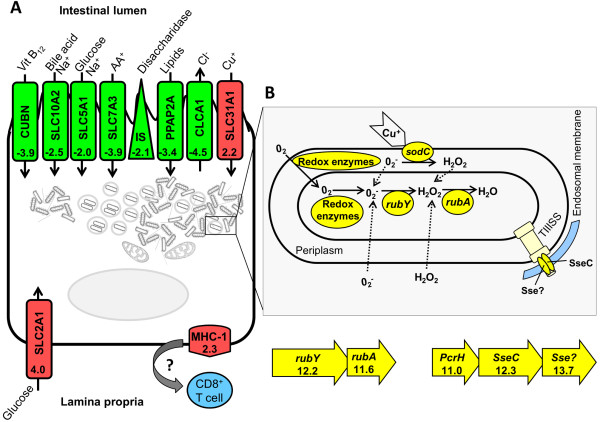
**Proposed model for host-pathogen interaction in porcine enterocytes infected with *L. intracellularis*. (A)** Infected enterocyte. Apical membrane exhibiting down-regulation of genes involved in nutrient acquisition and electrolyte secretion (green) and up-regulation of copper uptake protein (red). Basolateral membrane exhibiting up-regulation of glucose transporter (SLC2A1) and MHC class I genes. **(B)** Intracellular bacterium. High expression (yellow) of genes included in the oxidative stress protection system: redox enzymes, Cu-Zn superoxide dismutase (*sodC*) and rubrerythrin-rubredoxin (*rubY-rubA*) operon (Adapted from Lumppio et al. 2001). Moderate expression genes encoding the basal body of the type III secretion system (TIIISS) (light yellow) and high expression of TIIISS effector proteins (*PcrH*-*SseC*-*Sse?* operon). Genetic organization and gene expression (log_2 [RPKM]_) of the *rubY-rubA* and *PcrH*-*SseC*-*Sse* operons.

The functional clustering analysis revealed 14 differentially expressed genes associated with pro- and anti-apoptotic events (Table 3). All seven genes related to anti-apoptotic events were up-regulated in infected cells. Among the pro-apoptotic genes, four were down-regulated and three up-regulated. Interestingly, two of these pro-apoptotic genes that are also involved in the cellular immune response against intracellular pathogens (Signaling lymphocytic activation molecule 7 and Tumor necrosis factor ligand 10) were down-regulated. On the other hand, significant up-regulation of genes encoding the major histocompatibility complex class I (MHC-I) was identified in *Lawsonia*-infected enterocytes (Figure 4A).

### Nutrient acquisition and electrolyte secretion

Consistent down-regulation of numerous genes expressed in the apical membrane of enterocytes that are involved in nutrient acquisition was observed in *Lawsonia*-infected cells (Figure 4A). These membrane transporters are involved in the absorption of carbohydrates (sodium/glucose co-transporter and sucrase-isomaltase), amino acids (cationic amino acid transporter), bile acid (sodium/bile acid co-transporter), lipids (lipid phosphate phosphohydrolase) and Vitamin B_12_ (cubilin receptor). Additionally, the intracellular infection also affects the electrolyte secretion by decreasing expression of the chloride channel gene (CLCA1). The reduction of both nutrient acquisition and electrolyte secretion indicates that *L. intracellularis* may be able to prevent cell differentiation in immature enterocytes.

In contrast to the down-regulation of genes expressed on the apical membrane, the gene encoding the glucose transporter 1 (also known as solute carrier family 2, glucose transporter member 1) was highly up-regulated in infected enterocytes. This transporter was the third highest expressed gene (Additional file 2: Table S3) and its expression also has been reported on the basolateral membrane of human enterocytes *in vitro*[25] and rat jejunum *in vivo*[26]. Additionally, significant up-regulation of the high-affinity copper uptake protein (CTR1) involved in copper absorption was also found in infected enterocytes (Figure 4A).

### Host-pathogen interaction

The transcriptional landscape of the intracellular bacteria was determined by classifying all genes with at least one mapped read into one of three levels of expression: low, moderate and high. As expected, genes encoding ribosomal-related proteins were the functional category most associated with the mapped reads and the majority of these genes exhibited moderate to high expression. The *L. intracellularis* gene expression was characterized and discussed based on the most highly expressed bacterial genes (Table 1).

The functional categories of bacterial genes that were highly expressed included those involved in protein folding (e.g. *groES*-*groEL* operon and chaperone *dnaK*) and biosynthesis (e.g. elongation factor Tu), oxidative stress (e.g. Cu-Zn superoxide dismutase, *rubY*-*rubA* operon and dioxygenases), secretion system effector-related proteins (*PcrH*-*SseC*-LI1159 operon) and various hypothetical proteins. Since we were not able to distinguish free bacteria in the cell cytoplasm from those organisms within cell endosomes, the host-pathogen interaction was discussed considering both scenarios. Figure 4B illustrates the biological activities of the bacterial proteins encoded by highly expressed genes considering the intracellular microenvironment of *Lawsonia*-infected enterocytes.

While redox enzymes catalyze reduction of O_2_ derived from the cell cytoplasm, Cu-Zn superoxide dismutase C (*sodC*) and rubrerythrin-rubredoxin operon (*rubY*-*rubA*) neutralize reactive oxygen species (O_2_^-^ and H_2_O_2_) generated in the endosome or from the reduced O_2_ molecule (Figure 4B) [27]. An operon genetically related to the *Salmonella* pathogenicity island 2 (SPI2) composed of three genes, a chaperone (*PcrH*), an effector protein (*SseC*) and a hypothetical protein LI1159 (referred as *Sse?*) was highly expressed (Figure 4B). Interestingly, genes encoding the type III secretion system (TIIISS) apparatus whereby these effector proteins would be delivered to the cell cytoplasm showed only moderate expression (data not shown) and were located downstream of the SPI2-related operon on the *L. intracellularis* chromosome.

Among the ten genes most highly expressed by the intracellular bacterium, five represented hypothetical proteins. A summary of the predictive analyses evaluating their structures and biological functions are described in the supplemental material (Additional file 4: Table S4). Two different families of proteins were predicted for the gene locus LI0447 (aminomethyltransferase beta-barrel and growth factor receptor domain). Additionally, transmembrane proteins (porin and autotransporter), proline isomerase, extracellular protease and secretory factor were also identified based on predictive motifs.

## Discussion

The present study used LCM technology to microscopically dissect enterocytes from pigs experimentally infected with *L. intracellularis* and to characterize the cell and the bacterial gene expressions using high throughput sequencing (RNA-seq). The results demonstrated the usefulness of coupling LCM and RNA-seq techniques to study the host-pathogen interaction in a specific cell population present in a heterogeneous tissue. The simultaneous evaluation of the gene expression changes in both the host and the pathogen has been recently designated dual RNA-seq [28]. The challenge in studying both sets of transcriptional profiles from a common sample arises mainly because of the extensive variety of the bacterial organisms regarding their genomic compositions (e.g. %CG contents) and the particular characteristics of each infectious process at the cellular level. Since the abundance of eukaryotic RNA is significantly higher compared to the prokaryotic RNA in an infected cell, a sufficient number of bacteria per host cell followed by an unbiased RNA amplification step are crucial for studying the cell-bacteria interactions [28]. In light of this, we established the endpoint of our study (21 days PI), based on the chronological course of the *L. intracellularis* infection previously described [17,29]. This previous study demonstrated a progressive increase in the number of *L. intracellularis* in the feces of infected pigs from day two (10^2^) through day 22 (10^6^) post-infection. Confirming these previous observations, our study showed at 21 days PI, numerous bacteria in proliferative enterocytes indicating the active stage of the infectious process. We hypothesized that the disease at this point was approaching its peak when the intracellular bacteria were exhibiting their virulence factors and the host cells were responding to the pathogen at appropriate levels to allow us to evaluate the changes in gene expression. We believe that earlier endpoints would not provide a sufficient amount of RNA to be recovered and later stages of the disease would not represent the logarithmic phase of the bacterial growth. Additionally, the increasing presence of *L. intracellularis* within the lamina propria along the disease progression would also reduce the number of bacteria in enterocytes. In addition to the limitation of having only one time point, our study also could not evaluate the role of soluble proteins and growth factors secreted into the extracellular matrix of the lamina propria and their influence in the enterocyte proliferation and differentiation.

A previous chronological analysis using microarray technology evaluated the host response of fibroblastic cells *in vitro* at three time points (24, 48 and 72 hours PI) [30]. Altered transcription of genes related to cell cycle and cell differentiation were described [30]. However, cellular proliferation which is the main phenotypic characteristic of the *L. intracellularis* infections *in vivo* has not been reproduced in *in vitro* models [14,18]. Furthermore, the transcriptional response of mesenchymal cells which are not the natural target cells for *L. intracellularis* needs to be interpreted with caution.

A single time point analysis of the host gene expression profile using intestinal tissues from pigs naturally affected with PE was recently described [31]. Although this study provided an interesting snapshot of the transcriptional host response, it used field cases of diarrhea where the samples were co-infected with porcine circovirus type 2 which may be a confounding factor in the evaluation of the expression of genes especially related to the immune response [31]. Additionally, the microarray was performed using entire intestinal tissues. As a result, the specific characterization of the transcriptional host response was impaired by either the heterogeneity of the cell population included in the intestinal tissues or the virus infection. By applying the LCM technique to isolate ileal enterocytes in our study, we confirmed the identification of several genes specifically expressed in the intestinal epithelium of the ileum (e.g. ileal sodium/bile acid cotransporter) corroborating with a recent comprehensive study describing the gene expression atlas of the domestic pig [32].

Gene network and pathway analyses revealed that protein biosynthesis and activation of transcription were the host cellular events most associated with the genes differentially expressed in *Lawsonia*-infected enterocytes (Figure 3A). In agreement with these molecular findings, ultrastructural studies *in vivo* using electron microscopy identified the numerous bacteria occupying the apical cytoplasm of infected enterocytes which was otherwise composed almost entirely of free ribosomes and scattered mitochondria [29,33]. These morphological findings associated with our molecular results suggest a global increase in the cell metabolism in response to *L. intracellularis* infection. Taking the host cell cycle events into consideration, an increase in protein synthesis and transcription is required during the G_1_ phase in order to prepare the cell for subsequent division [22]. Interestingly, this protein synthesis network was also associated with cell proliferation at an early stage of infection with human immunodeficiency virus [34].

The differential expression of cell cycle-associated genes identified by the functional clustering analysis (Table 3) showed specific activation of Rho family genes (RhoA, RhoB and Rho GTPase). These molecules play a key role in carcinogenesis through their aberrant activation which results in cell proliferation [23]. Rho family proteins specifically act on the G_1_-checkpoint of the cell cycle when the transition to commit the cell to the proliferative stage occurs. If the signals responsible for promoting this transition are not present then the cells enter into the non-proliferative phase (G_0_) [35]. Additionally, a gene encoding Rho GTPase was also highly up-regulated during *L. intracellularis* infection *in vitro*[30], suggesting that exacerbated activation of the G_1_ phase is an important mechanism involved in the proliferative changes induced by *L. intracellularis* in infected enterocytes.

In addition to their roles in cancer development, Rho proteins are also pathologically activated by bacterial toxins also known as cyclomodulins [1,35,36]. Cytotoxic necrotizing factor (CNF) found in uropathogenic *Escherichia coli*, *Pasteurella multocida* toxin (PMT) and dermonecrotic toxin of *Bordetella* spp. act directly on Rho family proteins to bring about their irreversible activation [37]. Furthermore, CNF-expressing *E. coli* establish a persistent intracellular infection in the urogenital tract and suppress apoptosis by affecting the transcription levels of Bcl-2 family genes [37]. It is thought that apoptosis inhibition in target cells may favor bacterial persistence at the epithelium surface, thereby favoring bacterial replication and spread inside the host cell [38]. Although our study showed a predominant activation of anti-apoptotic-related genes compared with pro-apoptotic events, the Bcl-2 gene was not significantly activated in infected cells (log_2_-fold change = 1.06). Therefore, other mechanisms may be involved in the predominant activation of anti-apoptotic genes identified in the present study.

The contribution of apoptotic mechanisms for the pathogenesis of *L. intracellularis* infections has been speculated over the years and still needs to be elucidated. Initially, a temporary reduction in apoptosis was hypothesized to be an important mechanism involved in the cell proliferation [29]. Later studies suggested an increase in apoptosis based on the Caspase-3 immunohistochemical staining [8,39]. One of these studies described the dynamic of the Caspase-3 staining through the chronological evaluation of experimentally-infected animals and showed variations on the pattern of Caspase-3 staining within different parts of the intestinal mucosa on day 19 PI [39]. Our study showed activation of the gene-encoding Caspase-3 (log_2_-fold change = 1.12) in infected enterocytes, but the level did not reach the parameters for statistical significance. Considering the complexity and dynamism of the apoptosis process, the stage of the infection at the cellular level may directly influence the apoptotic gene network. Therefore, an *in vitro* model displaying the proliferative phenotype would be an ideal starting point to address this question. Despite the reduced expression of these two immune response-associated genes identified in our study, we observed significant up-regulation of MHC-I genes in infected cells, indicating that *Lawsonia*-derived antigen is presented to the lamina propria through the basal membrane of infected enterocytes (Figure 4A).

Although the physiopathology of diarrhea in *L. intracellularis* infections remains to be elucidated [40], the significant down-regulation of numerous genes related to nutrient acquisition observed in the present study indicates that malabsorptive diarrhea represents the major mechanism involved in the poor performance and growth of affected animals. Supporting our observations, a lower absorption of glucose and electrolytes was reported using a hamster experimental model of PE [41]. The reduced expression of nutrient acquisition-related genes also indicates the lack of cell differentiation in infected enterocytes.

The deficiency in nutrient acquisition described above seems to contrast with the increase in the cell metabolism characterized by the activation of transcription and protein synthesis-related genes also described here. As proposed in the Figure 4A, the high expression of the glucose transporter SLC2A1 on the basolateral membrane appears to compensate for this lack of nutrient acquisition from the intestinal lumen, since it has been described as being expressed in the basolateral membrane of human enterocytes *in vitro*[25] and rat jejunum *in vivo*[26].

The only gene significantly up-regulated in infected cells which is involved in nutrient acquisition and is physiologically expressed on the apical membrane is the high-affinity copper uptake gene [42]. Copper is an essential metal used by eukaryotic cells as a biochemical cofactor especially during the process of oxygen reduction by cytochrome *c* oxidase which leads to the production of ATP [43]. The production of ATP by the eukaryotic host is crucial for metabolism of *Lawsonia* and two other groups of obligate intracellular bacteria (*Chlamydiales* and *Rickettsiales*). These organisms express ATP/ADP translocase which catalyze the exchange of bacterial ADP for host ATP allowing bacteria to exploit their hosts’ energy pool, a process referred to as energy parasitism [44,45]. In addition to this essentiality for host cells, an increase in copper uptake has also been reported as a defense mechanism against intracellular pathogens because of its toxic properties. Free intracellular copper can lead to oxidative stress whereby cycling of copper oxidation and reduction produce reactive oxygen species through the Fenton reaction [43]. Interestingly, our study identified *L. intracellularis* genes related to oxidative stress highly expressed within the host cytoplasm (Figure 4B).

Three to five percent of the sequence reads generated in our study also mapped against the *L. intracellularis* genome. Less than 0.1% of the sequence reads mapped against four bacterial species commonly found in both infected and non-infected groups, when screened for the presence of virus and other bacteria. While three of these species represent unculturable organisms (*Candidatus*) related to insect symbionts, one (*Propionibacterium acnes*) is part of the normal flora of the skin, oral cavity, large intestine, the conjunctiva and the external ear canal of humans [46]. The identification of the genus *Peptoniphilus*, found exclusively in the control group, may be due to human contamination during the experimental procedures, since it was reported in the oral cavity through the human microbiome project (NCBI accession: PRJNA52051).

*L. intracellularis* genes belonging to the oxidative stress protection were the functional cluster most associated with genes highly expressed in the host cytoplasm. Although this fastidious organism requires a strict microaerophilic environment for cultivation in cell culture, *Lawsonia* is often located close to the cell mitochondria, where the transport of oxygen is continuous due to the oxidative phosphorylation [47]. Additionally, this intracellular location is important to exploit the host energy pool by exchanging bacterial ADP for host generated ATP, as discussed earlier. Based on this scenario, our study demonstrated that *L. intracellularis* displays a sophisticated mechanism to survive in this microenvironment by coping with the oxidative stress and potentially incorporating intracytoplasmic copper taken by the host to combat the intracellular infection (Figure 4A).

Our study identified high expression of an operon (*PcrH*-*SseC-Sse*) genetically related to the SPI2. The proteins included within this operon act as a translocon attached to the phagosomal membrane to allow translocation of effector proteins into the cell cytoplasm [48]. We hypothesize that this operon was highly expressed by *L. intracellularis* organisms in the cell endosome (Figure 4B).

We found five genes encoding hypothetical proteins among the ten highest expressed by *L. intracellularis* (Table 1). From the host gene expression, we identified significant activation of Rho family genes which are crucial for the progression of the G_1_ phase of the host cell cycle and are targeted by bacterial toxins [1]. Taken together, this evidence suggests the presence of an unrecognized cyclomodulin encoded by those highly expressed genes of *L. intracellularis*. Our results form the basis for future studies focusing on the specific properties and functions of these genes.

## Conclusions

The present study established a method integrating laser capture microdissection and RNA-seq technology to characterize both the host and the bacterial gene expressions *in vivo*. This methodology was applied to investigate the proliferative changes induced by an obligate intracellular pathogen, *Lawsonia intracellularis*, in intestinal crypt cells. The proliferative changes in infected enterocytes were associated with deregulation of the G_1_ phase of the host cell cycle based on the activation of transcription, protein synthesis and Rho family genes [22,24]. The ability of *L. intracellularis* in preventing enterocyte differentiation and maturation was proved by the consistent down-regulation of apical membrane transporters related to nutrient acquisition (Figure 4A). The gene encoding copper uptake protein was the only apical membrane transporter activated in the *Lawsonia*-infected cells indicating a host defense mechanism by inducing copper toxicity [43]. High expression of the Cu/Zn superoxide dismutase (*sodC*) gene by *L. intracellularis* suggests that the intracellular bacteria incorporate intracytoplasmic copper to cope with oxidative stress. In addition to *sodC*, the intracellular bacteria showed high level of expression of a sophisticated oxidative protection mechanism which involves redox enzymes and rubrerythrin-rubredoxin operon (Figure 4B). Finally, Rho genes expressed by the host and associated with the high expression of bacterial genes encoding hypothetical proteins implies a potential role of unrecognized bacterial effector proteins that modulate eukaryotic cell cycle (cyclomodulins) in the pathogenesis of PE.

## Methods

### Preparation and quantification of *L. intracellularis* inoculum

The porcine pathogenic *L. intracellularis* strain PHE/MN1-00 was previously isolated and continuously grown in cell culture using murine fibroblast-like McCoy cells (ATCC CRL 1696) for 10 passages [19]. The bacteria were then pelleted, suspended in sucrose-potassium glutamate (pH 7.0; 0.218 M sucrose, 0.0038 M KH_2_PO_4_, 0.0072 M K_2_HPO_4_ and 0.0049 M potassium glutamate) solution with 10% fetal bovine serum and stored at −80°C until the day of infection. The number of *L. intracellularis* organisms was assessed by direct counting after immunoperoxidase staining [49] and by quantitative PCR (qPCR), as described elsewhere [50].

### Experimental infection

Twelve Duroc-Landrace cross pigs at 3-weeks-of-age were divided into two groups: infected and control (n = 6/group). The animals were obtained from a herd with no history of PE and each treatment group was housed in a different pen. Prior to the study, blood and fecal samples from all animals were collected and tested for *L. intracellularis*-specific antibodies by immunoperoxidase monolayer assay (IPMA) and for the presence of *Lawsonia* DNA in the feces in order to confirm their negative status [11,49]. The animals were allocated in the isolation barns at the College of Veterinary Medicine of the University of Minnesota and fed with non-medicated nursery feed and water ad libitum. All procedures were approved by the Institutional Animal Care and Use Committee of the University of Minnesota. The infected group was orally inoculated with 30 ml of *L. intracellularis* culture at 10^8^ organisms per ml. The non-infected group was orally treated with sodium-phosphate-glutamate (SPG) solution. Fecal samples were collected every other day and analyzed by qPCR for the presence *L. intracellularis* DNA [11].

### Tissue collection and preservation

All animals were euthanized 21 post-infection (PI) approaching the peak of the clinical disease, as described previously [20]. Immediately after euthanasia, the ileal mucosa was washed three times with PBS solution containing 2.0 U/μl RNAse inhibitor (Roche Applied Science) [51]. A total of six tissue samples from each animal were collected, placed into plastic cryomold cassette (Tissue-Tek® Sakura Finetek) in longitudinal orientation and embedded in optimal cutting temperature (OCT) compound (Tissue-Tek® Sakura Finetek). The samples were then placed onto blocks of dry ice until the tissues and OCT were frozen to a solid white. All samples were stored at −80°C until use.

### Laser capture microdissection

Five serial 8-μm frozen sections were cut at −20°C using RNAse-free blades and mounted on StarFrost® RNAse-free slides (Fisher Scientific). The first and fifth cryosections (reference slides) were evaluated by immunohistochemistry (IHC) using the streptavidin method with polyclonal antibodies specific for *L. intracellularis*[52]. In these two reference slides the levels of infection were assessed based on the amount of positively labeled antigen present in the intestinal sections: Grade 0 (−) = no positive antigen labeled; Grade 1 (+) = one isolated focal area of antigen labeled; Grade 2 (++) = multi-focal areas of antigen labeled; Grade 3 (+++) = majority of the mucosa has positive antigen labeled; and Grade 4 (++++) = all of the mucosa has positive antigen labeled [17]. The series of slides containing the two flanking pairs of reference slides most severely infected (grade 3 or 4) was selected for LCM from each animal. Similar anatomic portions of the ileum were collected from the control group and stained by IHC to confirm the negative status of the samples. One non-infected series of cryosections was selected for LCM from the similar anatomic portion of those selected in the infected group.

The LCM procedures were performed in the second, third and fourth serially-cut slides using Histogene^TM^ staining (Life Technologies). Briefly, the specimens were thawed at room temperature for 30s, fixed in nuclease-free 75% ethanol, rehydrated in nuclease-free water for 30s, stained with HistoGene® solution for 20s and rinsed in water for 30s, then dehydrated by sequential immersion into 75%, 95% and 100% ethanol for 30s each. All microdissection procedures were performed at the College of Biological Sciences Imaging Center at the University of Minnesota using PixCell® II System (Life Technologies). Single-cells were captured from the slides using CapSure LCM caps (Life Technologies) with laser spot diameter 7.5 μm, power 60 mW and duration 650 μs. Approximately, one thousand cells were captured from the bottom to the upper part of the intestinal crypts (Figure 1A and 1B) in each of the three stained slides in approximately ten minutes, incubated at 42°C for 30 min in 50 μl extraction buffer and stored at −80°C. The microdissected cells from each of the three serially-cut slides were pooled and used for the RNA extraction.

### RNA isolation and amplification

Total RNA was isolated from microdissected tissues using a PicoPure™ Kit (Life Technologies) according to the manufacturer’s instructions. Genomic DNA was digested using DNase I (Qiagen). RNA samples were then assessed with a NanoDrop ND-8000 Spectrophotometer and Agilent Bioanalyzer 2100. Amplified cDNA was prepared from 100 ng of total RNA using the Ovation® RNA®Seq System V2 (Nugen®), following the manufacturer’s instructions. The amplification was initiated at the 3’ end as well as randomly throughout the sample which allows the amplification of eukaryotic and prokaryotic RNA transcripts. The amplified cDNA generated was then purified using MinElute Reaction Cleanup Kit (Qiagen) for library preparation.

### Library preparation and sequencing

The library preparation and sequencing were conducted in the core facility of the Biomedical Genomics Center at the University of Minnesota. Briefly, 100 ng of the amplified cDNA was fragmented, ends repaired, enriched by PCR and validated using High Sensitivity Chip on the Agilent2100 Bioanalyzer. Following quantification of the cDNA generated for the library using PicoGreen Assay, the samples were clustered and loaded on the Illumina® Genome Analyzer GA IIx platform which generated on average 22,136,064 paired reads of 100 bp. Base calling and quality filtering were performed following the manufacturer’s instructions (Illumina® GA Pipeline).

### Filtering and mapping of sequence reads

Quality control, trimming and mapping were performed in the Galaxy platform [53]. Initially, FastQC tool was applied to the raw sequence data followed by FastQ Trimmer [54]. As a result, 15 base pairs were trimmed from the 5’end and ten from the 3’end of each raw sequence read. Sequences containing 75 bp and a phred quality score of more than 20 were used in the gene expression analysis [55]. Using Bowtie short read aligner with no more than two mismatches, the filtered sequences were mapped onto the pig genome *S. scrofa* 10.2 and the *L. intracellularis* isolate PHE/MN1-00 reference genome, both obtained from the National Center for Biotechnology Information (NCBI) database [56]. The number of reads mapped within each annotated transcript was calculated in order to estimate the level of transcription for each gene. Cufflinks tool was used to estimate the relative abundances of the transcript reads for each gene [57]. For comparing the levels of gene expression between infected and non-infected enterocytes and characterizing the abundance of *L. intracellularis* transcripts expressed in the host cytoplasm, the read counts were normalized based on the number of reads per kilobase of coding sequence per million mapped reads (RPKM).

In order to determine any potential co-infection associated with the intracytoplasmic *L. intracellularis*, the sequence reads were screened against 4,279 viral sequences available at NCBI database. Additionally, MetaPhlAn (Metagenomic Phylogenetic Analysis) was performed to identify other bacterial genome sequences in the experimental samples [58].

### Differential gene expression

The differential gene expression between infected and non-infected enterocytes was assessed using the CuffDiff tool. This tool defines the total read count for each gene by combining the expression data from all the replicates in each experimental group and testing for differential regulation in expressed transcripts present in at least three replicates. RPKM values were expressed in log_2_ (RPKM) to allow the statistical comparison. As a result, log_2_-fold change in abundance of each transcript was obtained by log_2_ (RPKM [infected]/RPKM [non-infected]). *P*-values were calculated and adjusted for multiple comparisons using false discovery rate (FDR) [59]. Significant differential expression was determined in genes with FDR-adjusted p-values < 0.05 and fold change ± 2.

### Functional gene ontology and pathway analysis

Biological functions and interactions of the genes differentially expressed were determined using the database for annotation visualization and integrated discovery (DAVID v6.7) and the Ingenuity pathway analysis (IPA) (Ingenuity® Systems). While the DAVID knowledge database was used for functional clustering, the mammalian knowledge database of the IPA was used for pathway and network analyses of the differentially expressed genes.

Predictive analysis regarding the ontology and motifs of gene-encoding hypothetical proteins highly expressed by *L. intracellularis* were performed using Kyoto Encyclopedia of Genes and Genomes (KEGG) database. Additionally, Phyre^2^ (Protein Homology/analogY Recognition Engine) [60] and ConFunc (Protein Function Prediction Server) [61] software were used to predict their biological functions and three-dimensional structure.

### Reverse transcription PCR

In addition to the assessment of RNA quality based on the bacterial and eukaryotic ribosomal RNAs using Agilent Bioanalyzer 2100, one-step RT-PCR was applied to the RNA extracted from microdissection tissues in order to evaluate the quality of bacterial mRNA for RNA-seq analysis. The one-step RT-PCR (Qiagen®) was used with specific primers (Additional file 3: Table S1) targeting three housekeeping genes of *L. intracellularis*.

Quantitative RT-PCR (qRT-PCR) was performed for validating the expression data identified by RNA-seq using a specific set of genes from the host and bacterium (Additional file 3: Table S1). RNA samples were synthesized to first-strand cDNA using SuperScript® II RT (Invitrogen). Duplicate qRT-PCR reactions from each primer probe set were validated by five serial dilutions of cDNA on the ABI7900HT instrument (Applied Biosystems). After validation, quantitative PCR was performed in duplicate using 15ng of cDNA per sample with the following conditions: 60°C for 2 min, 95°C for 5 min and 45 cycles (95°C/10 sec and 60° for 1 min). The differential expression for the host gene expression were described as relative fold-change between infected and non-infected enterocytes based on Ct values (2^-∆∆Ct^), as previously described [62]. The suitability of four porcine housekeeping genes (Beta actin, Cyclophilin A, Hypoxanthine phosphoribosyltransferase, Ribosomal protein L4) for qRT-PCR analysis was evaluated.

Based on consistent Cyclophilin A gene expression throughout the biological replicates, this gene was used to normalize the expression data. For the bacterial gene expression, averages of relative transcriptional levels normalized by the 30S ribosomal gene were calculated and compared to the RNA-seq expression levels. A linear regression model was used to evaluate the correlation between average RPKM and qRT-PCR data, as described previously [63].

## Competing interests

The authors declare they have no competing interests.

## Authors’ contributions

FAV contributed to the study concept, design, acquisition and analysis of data, writing of manuscript. DNF contributed to the study concept, design, writing of manuscript and study supervision. CJG contributed to the study concept, design, acquisition and analysis of data, writing of manuscript and study supervision. All authors read and approved the final manuscript.

## Supplementary Material

Additional file 1: Table S2 Sequencing and mapping of each biological replicate. Click here for file

Additional file 2: Table S3 Genes differentially expressed by enterocytes infected with *L. intracellularis*. Click here for file

Additional file 3: Table S1 Primers used for one-step RT-PCR and for validation of RNA-seq expression data by quantitative reverse transcriptase PCR assay. Click here for file

Additional file 4: Table S4 Predictive analysis of the highest expressed *L. intracellularis* genes encoding hypothetical proteins. Click here for file
